# COVID-19 Vaccine Hesitancy

**DOI:** 10.9745/GHSP-D-22-00043

**Published:** 2022-02-28

**Authors:** Doug Storey

**Affiliations:** a Johns Hopkins Center for Communication Programs, Baltimore, MD, USA.

## Abstract

Improving access to accurate information on vaccines and vaccination, increasing trust in reliable information sources, and counteracting misinformation can go a long way toward improving vaccination decision making.

See related article by Kulkarni et al.

As access to the coronavirus disease (COVID-19) vaccination expands around the world, attention has turned to the factors that influence vaccine acceptance and hesitancy.^1^^–^^4^ For example, the COVID Behaviors Dashboard (https://covidbehaviors.org/),^5^ a collaboration between Facebook/META, Johns Hopkins University, Carnegie Mellon University, and the University of Maryland, tracks reasons for vaccine hesitancy in 115 countries around the world.

The percentage of unvaccinated people who simply don’t like vaccines and reject them outright is relatively small—about 14% globally.^5^ Some people experience structural barriers to vaccination,^6^ such as eligibility, difficulty making or getting an appointment, difficulty taking time off from work or school or needing childcare to go for vaccination, distance to a vaccination site, and language barriers. Naturally, the challenges people face vary somewhat from country to country and can change over time.

Other common reasons for vaccine hesitancy are psychosocial in nature and more directly amenable to communication efforts to overcome them. These reasons include concern about side effects, uncertainty about vaccine effectiveness, wanting to wait and see if vaccines are safe, and perceived behavioral norms among one’s peers. While these psychosocial factors are common in many places, they are also shaped by local culture and social conditions and must be studied locally to develop tailored approaches to vaccine communication. For example, patterns of social interaction differ resulting in localized normative perceptions, real and perceived risk of COVID-19 transmission vary, and sources of information differ in their trustworthiness. In this issue of *GHSP*, Kulkarni et al.^7^ examine the roles that different information sources have in uptake of the third dose of diphtheria-pertussis-tetanus-hepatitis B-*Haemophilus influenzae* type-b-pentavalent vaccine. Such variations in these factors indicate a need for continued analysis of how people think about vaccines and vaccination and the social influences that affect their decisions about getting vaccinated.

Where and how people get COVID-19 information affects what they think about the disease and how to respond. Formative research to inform COVID-19 vaccination communication efforts should be asking people who have been vaccinated questions such as:

- What made you decide to get the vaccine?
- What do you consider to be the benefits of getting vaccinated?
- Was there anything that made it hard for you to get vaccinated?
- How did you overcome that challenge?
- Where did you get the information that helped you make the decision and get vaccinated?

People who have not yet been vaccinated should be asked the following questions:

- What are some of the reasons you have not been vaccinated yet?
- How likely do you think it is that you might become infected with COVID-19?
- How serious do you think it would be if you did become infected with COVID-19?
- What proportion of your friends and family has been vaccinated?
- Where do you get information that helps you decide whether to get vaccinated or not?

The COVID-19 pandemic is global and has connected us in ways we have rarely if ever seen before. Although still not universal, rapidly expanding access to mobile technologies in all parts of the world makes it easier to access information and connect with others across time and distance at unprecedented speeds. As a result, we vicariously share experiences (and trauma) with people who are otherwise quite different from us. Sadly, the same technologies can also spread misinformation and enable close-minded echo chambers and discriminatory communities to form and persist.^8^ People develop attitudes and beliefs through interaction with others and through what they see and hear in the media, increasingly through online and social media, even in more remote parts of the world. Furthermore, they make choices based in part on what they see their peers and other community members doing and on what they perceive to be approved or disapproved by people around them, so the symbolic environment that surrounds them can be very influential.

The COVID Behaviors Dashboard also opens a window on these dynamics of trust and information use. Information sources that are trusted generally exert more persuasive influence over people who rely on them, but trust does not necessarily result in better health outcomes. Trusting in a source that contains misinformation may pose real danger to personal and public health. According to the Dashboard data, patterns of media use and trust vary somewhat from country to country, but globally the sources of information people report relying on the most are government health authorities and journalists. However, those 2 sources differ considerably in terms of reported trustworthiness.^5^ The least trusted sources of information tend to be journalists, religious leaders, and politicians, while scientists and health experts, including international agencies like the U.S. Centers for Disease Control and Prevention and the World Health Organization, and local health workers and government health authorities, enjoy higher levels of trust. Fortunately, sources that engender low levels of trust tend to have lower levels of exposure, but people who do trust those sources are more susceptible to the misinformation they carry. Communication planners seeking to promulgate accurate information about COVID-19 should carefully analyze the relationship between trustworthiness and exposure to identify the communication channels that are **both** trusted **and** reach the greatest numbers of people.

From a public health perspective, managing the symbolic environment to improve access to accurate information, to improve trust in trustworthy information sources, and to counteract misinformation can go a long way toward improving vaccination decision making.

## Notes

**Peer Reviewed**
